# Simultaneous Estimation of Four Antitussive Components from Herbal Cough Syrup by HPTLC

**DOI:** 10.1155/2014/976264

**Published:** 2014-10-28

**Authors:** Sharada L. Deore, Payal S. Jaju, Bhushan A. Baviskar

**Affiliations:** Government College of Pharmacy, Kathora Naka, Amravati 444604, India

## Abstract

A new simple, rapid, selective and precise high performance thin layer chromatographic (HPTLC) method has been developed for simultaneous estimation of vasicine, glycyrrhizin, eugenol, and cineole in herbal cough syrup. The retention factors of vasicine, glycyrrhizin, eugenol, and cineole are 0.53, 0.44, 0.75, and 0.77, respectively. Chromatography was performed on 60F_254_ percolated TLC plate using n-hexane : ethyl acetate : glacial acetic acid (8.5 : 1.0 : 0.5 v/v/v). Methods are validated according to ICH guidelines and can be adopted for the routine analysis of vasicine, glycyrrhizin, eugenol and cineole in herbal cough syrup.

## 1. Introduction

Herbal cough syrup is a pharmaceutical dosage form used to treat coughing and related conditions. Many of these herbal or ayurvedic cough syrups are incorporated with cough suppressants or expectorants from vasaka, liquorice, tulsi, ginger, kantkari, black pepper, camphor, and many more [1]. Out of these plants, constituents from vasaka, liquorice, tulsi and ginger are chosen for simultaneous estimation.

Vasaka is perennial, evergreen shrub of biological source* Adhatoda vasica* belonging to family Acanthaceae. It contains quinazoline alkaloids like vasicine and vasicinone and also essential oil. Vasicine present in the leaves possesses respiratory stimulant activity and induces bronchodilation as well as relaxation of the tracheal muscle. It offered significant protection against histamine induced bronchospasm [2]. Liquorice is root of* Glycerrhiza glabra* belonging to family Leguminosae. It contains majorly triterpenoid saponin glycyrrhizin and few flavonoids. Glycyrrhizin exerts antitussive effect by stimulating salivation and inducing a more frequent swallowing reflex [3]. Tulsi is evergreen plant of* Ocimum sanctum *belonging to family Lamiaceae. It contains volatile oil 0.4–0.8% comprising eugenol and beta-caryophyllene and many other monoterpenes. Eugenol shows an antitussive effect by central action mediated through both opioid and GABAergic system [4]. Ginger is dried as well as fresh rhizomes of* Zingiber officinalis* belonging to family Zingiberaceae. It contains 1–4% volatile oil containing cineole, zingiberene, borneol, and resins like gingerol and shogaol. Cineole shows antitussive effect by suppressing the cough reflex through direct action of cough centre in the medulla [5].

According to World Health Organisation's (WHO) standardisation guidelines, development and validation of analytical methods are needed to help in discovery, development, and marketing of quality herbal formulations. The literature [6–9] revealed that various TLC methods are available for estimation of vasicine, glycyrrhizin, eugenol, and cineole as individual component only; hence, in present work attempt has been made to develop simple, rapid, accurate, precise, and economical method for simultaneous estimation of all these four constituents in herbal cough syrup.

HPTLC is preferred over HPLC because HPTLC offers visual inspection throughout the process, less solvent consumption, multiple choices of fresh stationary phases, faster simultaneous estimation of many compounds with good analytical precision, and accuracy within less time.

## 2. Experimental

### 2.1. Reagents and Materials

**Figure 1:**
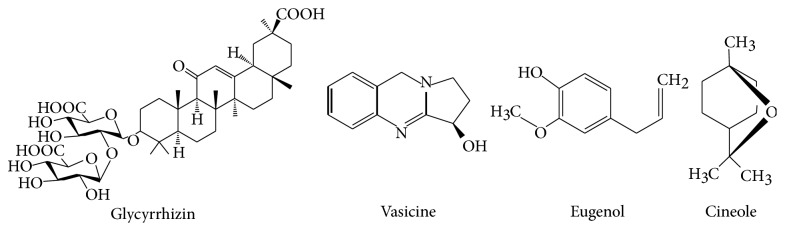


All chemicals and solvents used were of AR and HPLC grade (E. Merck, Mumbai, India). Marker standard vasicine, glycyrrhizin, eugenol, and cineole (Figure 1) were obtained as gift sample from Natural Remedies, Bangalore, India. TLC aluminium plates precoated with silica gel 60F^254^ (20 cm × 20 cm, 0.2 mm thick) were from E. Merck, Darmstadt, Germany.

### 2.2. Instrumentation

The method has been developed on Camag HPTLC system (Camag, Muttenz, Switzerland) consisting of twin trough chamber, Linomat V applicator, TLC scanner III, and WinCATS software version 1.4.4. Separation and identification of vasicine, glycyrrhizin, eugenol, and cineole were performed on aluminium backed silica gel 60F^254^ (20 cm × 10 cm of plate size, layer thickness 0.2 mm).

### 2.3. Chromatographic Conditions

**Figure 2:**
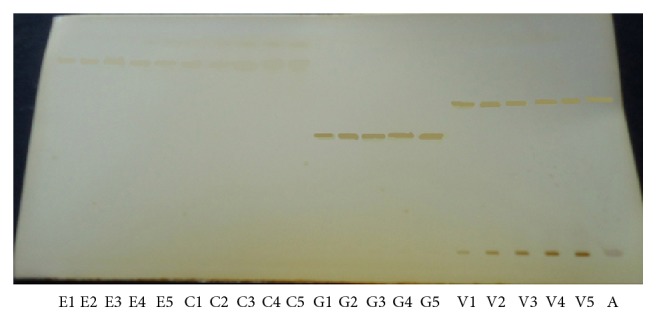
HPTLC plate showing simultaneous estimation of eugenol (E1–E5), cineole (C1–C5), glycyrrhizin (G1–G5), vasicine (V1–V5), and adulsa cough syrup (A).

Standard solutions of vasicine (99%), glycyrrhizin (98%), eugenol (98%), and cineole (98%) were prepared either separately or in a mixture at a concentration of 1 mg mL each in absolute methanol. Ultrasonication of mixture was required to ensure complete dissolution. The experiment was performed on a silica gel 60F^254^ (0.2 mm thickness) HPTLC plates (20 × 10 cm). Samples were applied by Linomat-V applicator to the plates as 4 mm bands with 4 mm distance. Many mobile phase compositions from solvents toluene, ethyl acetate, n-hexane, methanol, glacial acetic acid, and water were tried. Finally the plates were developed by the ascending technique, in a twin trough glass chamber with a stainless steel lid, using an optimised mobile phase composed of n-hexane : ethyl acetate : glacial acetic acid (8.5 : 1 : 0.5 v/v/v). The chamber saturation time was kept as 20 min. After development, plates were dried with a hot hair dryer; the spots which separated on plate were visualised when it was placed in iodine chamber and then scanned with a TLC Scanner, using WinCATS software in absorbance mode, with slit dimensions 3.00 × 0.45 mm. The detection wavelength 300 nm was selected. The Rf values for vasicine, glycyrrhizin, eugenol, and cineole were 0.53, 0.44, 0.75, and 0.77, respectively, as shown in Figure 2.

### 2.4. Quantification of Vasicine, Glycyrrhizin, Eugenol, and Cineole through Calibration Curve

All the standards were spotted in triplicate on 20 × 10 cm TLC plates for preparing six-point linear calibration curves. The calibration graph was plotted using the concentration versus average peak area at 300 nm for all standards. Peak area and concentration data were treated by linear least-squares regression analysis. Various standard dilutions of vasicine, glycyrrhizin, eugenol, and cineole were applied on TLC plate, developed, and scanned to know linearity range with reference to peak area.

### 2.5. Validation

As per ICH guidelines the method is validated for linearity, accuracy, precision, LOD, LOQ, ruggedness, and robustness [10].

(i) Linearity: standard stocks of 1 mg mL^−1^ of vasicine, glycyrrhizin, eugenol, and cineole were prepared and diluted to appropriate concentrations. The linearity was evaluated using linear least-squares regression analysis for generation of calibration curve. The regression equation with slope, intercept, and coefficient of correlation was calculated (Table 1).
(ii) Accuracy* (recovery)*: the accuracy of the method was assessed by spiking preanalyzed samples with known amounts of standard vasicine, glycyrrhizin, eugenol, and cineole solution and then reanalysed by the HPTLC method. The spiking was done at three different concentration levels and average percent recovery at each concentration level was calculated (Table 1).
(iii) Precision: the precision (%RSD) was determined by analysing standard solution of vasicine, glycyrrhizin, eugenol, and cineole over the entire calibration range for different days.
(iv) *Limit of detection*: the limit of detection (LOD) was determined using formula LOD = 3.3(SD)/*S*, where SD is standard deviation of response and *S* is average of the slope of the calibration curve. The minimum detectable limit was found to be ng/spot for vasicine, glycyrrhizin, eugenol, and cineole.
(v) Limit of quantification: the limit of quantitation (LOQ) was determined using formula LOQ = 10(SD)/*S*, where SD is standard deviation of response and *S* is average of slope of the calibration curve. The minimum quantified limit was found to be ng/spot for vasicine, glycyrrhizin, eugenol, and cineole. It was observed that other constituents present in the formulation did not interfere either with the peak of vasicine, glycyrrhizin, eugenol, or cineole. Therefore the developed method is specific.
(vi) Ruggedness of the method:it expresses the precision within laboratories variations like different analysts. Ruggedness of the method was assessed by spiking the standards 5 times with different analyst by using the same equipment.
(vii) Robustness of the method: by introducing small changes in the mobile phase composition, development distance, mobile phase volume, and duration of chamber saturation, the effects on the results were examined.

## 3. Results and Discussion

HPTLC method was optimized with view to quantify all four constituents in liquid herbal cough syrup. As far as individual estimation of vasicine, glycyrrhizin, eugenol, and cineole by chromatographic methods is concerned, a number of solvent systems have been reported. However, there has not been cited a single report for separation of vasicine, glycyrrhizin, eugenol, and cineole simultaneously in a single solvent system. n-Hexane : ethyl acetate : glacial acetic acid in ratio of 8.5 : 1.0 : 0.5 v/v/v shown good resolution for vasicine, glycyrrhizin, eugenol, and cineole with Rf value of 0.53, 0.44, 0.75, and 0.77, respectively. Well-defined spots were obtained after chamber was saturated for 20 min at room temperature. TLC plate was visualized when placed in iodine chamber without derivatization. The identity of vasicine, glycyrrhizin, eugenol, and cineole was confirmed by comparing chromatogram of standard vasicine, glycyrrhizin, eugenol, and cineole with that of extract and by comparing retention factor of reference with standard.

**Figure 3:**
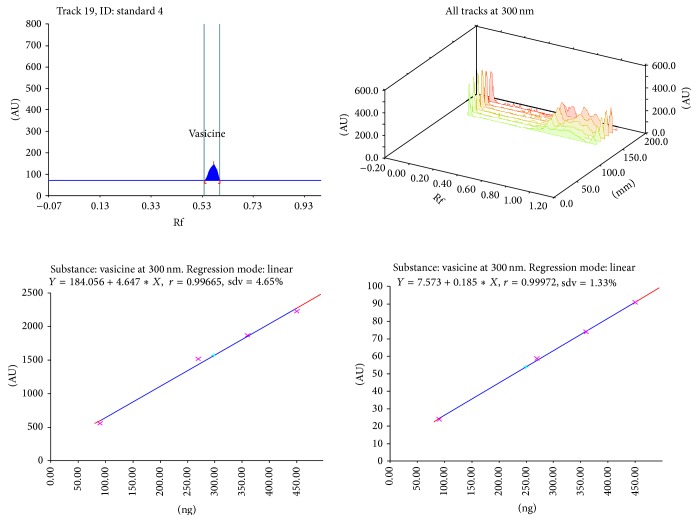
Chromatogram, calibration curve, and 3D display of vasicine.

**Figure 4:**
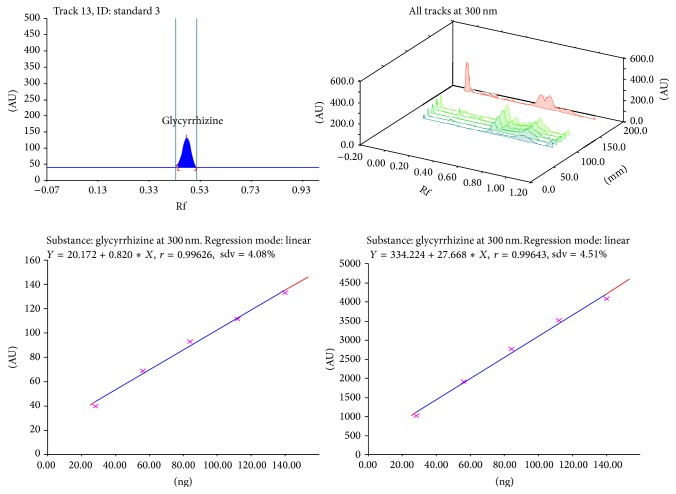
Chromatogram, calibration curve, and 3D display of glycyrrhizin.

**Figure 5:**
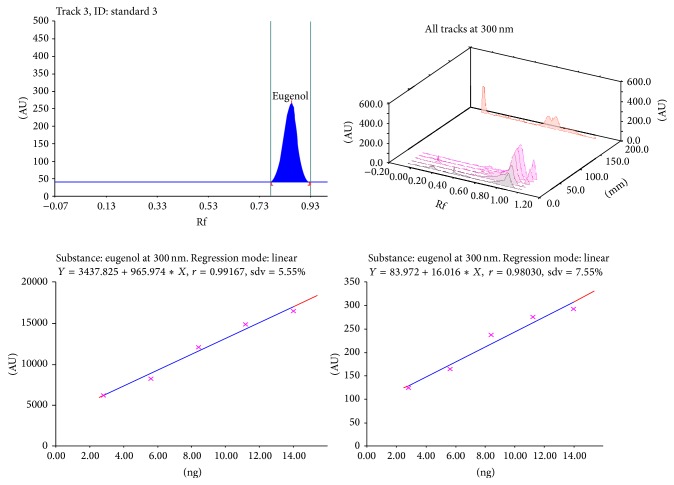
Chromatogram, calibration curve, and 3D display of eugenol.

**Figure 6:**
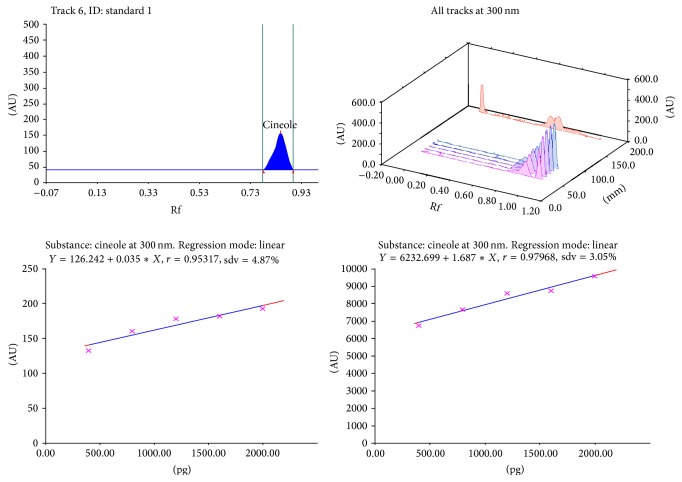
Chromatogram, calibration curve, and 3D display of cineole.

Calibration plot shown in Figures 3, 4, 5, and 6 indicates that the response is linear function of concentration in the range of 300–900, 1000–5000, 400–1000, and 2000–10,000 ng/spot for vasicine, glycyrrhizin, eugenol, and cineole, respectively. The correlation coefficient, intercept, and slope for vasicine, glycyrrhizin, eugenol, and cineole are given in Table 1.

As per validation results given in Table 2, lower %RSD (<2%) suggests precision of the method. The good recovery within acceptable limit indicates accuracy of method. The limit of detection (LOD) and limit of quantitation (LOQ) of vasicine, glycyrrhizin, eugenol, and cineole show adequate sensitivity of method. Good correlation of 0.99, 0.99, 0.99, and 0.97 was obtained between the standard and sample of vasicine, glycyrrhizin, eugenol, and cineole, respectively.

**Figure 7:**
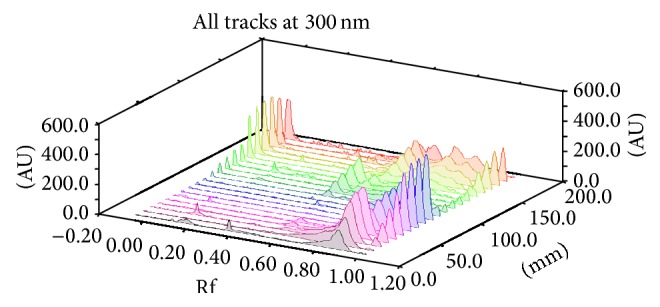
3D display of vasicine, glycyrrhizin, eugenol, and cineole in simultaneous estimation.

Ruggedness and robustness parameters confirmed that the method is able to withstand minor experimental changes (Table 2). It was observed that other constituents present in the formulations did not interfere with any of the peaks of all four constituents (Figure 7); therefore the method is specific.

## 4. Conclusion

Therapeutic efficacy, safety, and quality evaluation of herbal formulations is basic requirement of many regulatory authorities all over the world to grant its marketing approval and advanced analytical techniques are proving to be rapid and specific tool in the herbal drug research as well as in setting specific quality standards by their manufacturers. Hence, in the present work, for the first time, a rapid, simple, accurate, and specific HPTLC method for quantitative estimation of four antitussive constituents, that is, vasicine, glycyrrhizin, eugenol, and cineole present in herbal cough syrup, has been developed and validated. The present method could be part of routine quality analysis of any polyherbal cough syrups available in market despite interference of other constituents. Limitation like closeness of Rf values of cineole and eugenol is overcome by the use of HPTLC method instead of HPLC. Still future perspective involves better resolution with these two components along with separation and estimation of maximum antitussive components from herbal cough syrup by HPTLC.

## Figures and Tables

**Table 1:** Method validation parameters for quantification of vasicine, glycyrrhizin, eugenol, and cineole in herbal cough syrup by the proposed HPTLC method.

Validation parameters	Vasicine	Glycyrrhizin	Eugenol	Cineole
Correlation coefficient, *n* = 3	0.99	0.99	0.99	0.97
Linearity range (ng/spot), *n* = 3	300–900	1000–5000	400–1000	2000–10,000
Rf	0.53	0.44	0.75	0.77
Limit of detection (ng/spot), *n* = 3	45	105	60	225
Limit of quantification (ng/spot), *n* = 3	125	350	175	800
Instrumental precision (RSD), *n* = 6	0.58	0.48	0.35	0.47
Method precision (RSD), *n* = 6	0.72	0.59	0.42	0.58
Specificity	Specific	Specific	Specific	Specific
Robustness (RSD), *n* = 3	0.92	0.94	0.85	0.92

**Table 2:** Parameters used for ruggedness and robustness.

Sr. number	Parameter	Initial condition	Changed condition	Effect
1	Mobile phase	n-Hexane : ethyl acetate : glacial acetic acid (8.5 : 1 : 0.5)	n-Hexane : ethyl acetate : glacial acetic acid (8 : 1 : 1)	Very minute effect on the resolution, quantitative estimations, Rf, and peak area/height

2	Development distance	4 cm	3 cm	Very minute effect on the resolution, quantitative estimations, Rf, and peak area/height

3	Temperature	27 ± 3°C (winter)	35 ± 3°C (summer)	Affects greatly the resolution, quantitative estimations, Rf, and peak area/height

4	Tank saturation time	25 min	30 min	Very minute effect on the resolution, quantitative estimations, Rf, and peak area/height

5	Analyst	Analyst 1	Analyst 2	Very minute effect on the resolution, quantitative estimations, Rf, and peak area/height
